# Editorial: Advanced electrocatalytic materials for water electrolysis and fuel cells

**DOI:** 10.3389/fchem.2024.1373189

**Published:** 2024-02-01

**Authors:** Milutin Smiljanić, Branimir Grgur

**Affiliations:** ^1^ Department of Materials Chemistry, National Institute of Chemistry, Ljubljana, Slovenia; ^2^ Department of Physical Chemistry and Electrochemistry, Faculty of Technology and Metallurgy, University of Belgrade, Belgrade, Serbia

**Keywords:** electrocatalysis, hydrogen energy, water electrolysis, fuel cells, advanced materials

Advancing clean, affordable, and renewable energy is one of the key challenges of the modern world. Hydrogen has been labeled as the next-generation energy carrier able to replace fossil fuels on a large scale (Bockris, 2013). Production and utilization of green hydrogen can be achieved in electrochemical devices, namely water electrolyzers and fuel cells. The cores of these devices are catalytic materials needed for the efficient running of the occurring electrochemical reactions. Therefore, tailoring more active, durable, and cost-effective electrocatalysts plays a crucial role in the pursuit of a sustainable energy cycle.

Water electrolysis is a carbon-neutral technology for hydrogen production and relies on two electrochemical reactions, namely hydrogen evolution reaction (HER) and oxygen evolution reaction (OER). Efficient electrocatalytic materials for both HER and OER are needed to make the overall energy demand for hydrogen generation as low as possible. Platinum group metals (PGMs), namely Pt and Ir, in the form of small-sized nanoparticles dispersed over high surface area supports, are state-of-the-art catalysts for HER and OER, respectively. Despite the superior performance, the usage of PGMs is hampered by their scarcity and high price; therefore, the design of catalysts based on more abundant materials is highly desirable. In this context, transition metal-based catalysts stand out due to their feasible activity combined with abundance and economic viability. Cobalt (Co) based materials are known for their ability to catalyze both water-splitting reactions. Ahmed et al. prepared a hydrated cobalt phosphate-based carbon-nanofiber-supported material (Co_3_(PO_4_)_2_.8H_2_O/CNFs) using a hydrothermal approach. The presence of water in the obtained material was confirmed using several characterization techniques. Co_3_(PO_4_)_2_ × 8H_2_O/CNFs catalyst revealed enhanced HER activity in acid media making a significant approach to the Pt/C benchmark (*the Holy Grail of hydrogen electrocatalysis*). In terms of stability, the Co_3_(PO_4_)_2_ × 8H_2_O/CNFs catalyst revealed 24 h of fully stable operation, as confirmed by both electrochemical investigations and structural characterization. This novel composite with a flower-like structure benefited from high electrochemical surface area, high conductivity, and high exposure of edge sites in phosphates due to the vertical growth of hydrated phosphates, which altogether was found to be crucial for designing active electrode material.

Photo-electrocatalysis utilizes light energy to drive electrochemical reactions onto the electrode surfaces. The synergy between light absorption and electrocatalysis holds promise for numerous applications, one of the most attractive being related to hydrogen production by solar-driven water electrolysis (Hota et al., 2023). Moreover, photoelectrochemical (PEC) water splitting offers a renewable and efficient way to store the excess of intermittent solar energy in the form of hydrogen. Consequently, the development of electrode materials for PEC water electrolysis is of particular importance. Aleksić et al. reported a composite material consisting of ZnO and RuO_2_ as a bifunctional PEC catalyst for water-splitting reactions operating in both alkaline and acid electrolytes. It was shown that the addition of a small amount of RuO_2_ to ZnO (1:10 M ratio) significantly increased the absorption properties of ZnO and improved its photoelectrocatalytic properties for HER and OER. This enhancement was ascribed to a combination of improved electron transfer due to the metallic conductivity of RuO_2_, reduced number of oxygen vacancies, and increased number of heterojunctions (i.e. increased number of active sites).

State-of-the-art electrocatalytic composites imply small-sized metallic nanoparticles dispersed over convenient support, which is expected to provide high specific surface area, conductivity, and durability. Smiljanić et al. provided a summary of the most recent advances in the development of support materials, labeling carbon and non-carbon-based supports as the two main directions in the field. Carbon materials are benchmark supports in electrocatalysis thanks to their high specific surface area and high conductivity. Compared to classical carbons, graphene and its derivatives offer higher surface area and conductivity, higher *sp2* content, and fewer structural defects. Similar advantages are provided by carbon nanotubes and nanofibres, which benefit from their exceptional mechanical strength and tubular structure that facilitates mass transport. Andrić et al. reported a facile synthesis method to obtain CoPt alloy nanoparticles supported on different carbon-based materials, namely reduced graphene oxide, mesoporous graphitic carbon nitride, and Ketjen black. All three composites showed higher OER efficiency in alkaline media with respect to commercial CoPt/C catalysts, which was linked with enhanced charge transfer and effective utilization of the active sites during OER. CoPt/rGO composite exhibited the best catalytic OER activity with more than 10 times higher specific current densities compared to the CoPt/C analog. In terms of stability, CoPt nanoalloy supported on mesoporous graphitic carbon nitride showed superior properties, while (the most active) CoPt/rGO revealed quite unstable long-term operation. The observed durability issue needs to be addressed in future studies to make this material a suitable candidate for application as an OER catalyst, perhaps by doping with heteroatoms (such as B, N, P, and S) to improve the interaction between carbon supports and metallic nanoparticles. Furthermore, Smiljanić et al. labeled transition metal oxides (TMOs) as attractive alternatives for carbon in electrocatalysis due to excellent corrosion resistance and the possibility of inducing metal-support interaction (MSI). One of the most widely studied materials in this context is TiO_2_, which can be mixed with carbon to address its semiconducting nature. Alternatively, partial nitridation of TiO_2_ to form conductive TiON can be used to obtain advanced support for various electrocatalysts (Bele et al., 2019). Other TMOs with interesting properties include SnO_2_ and WO_3_; similarly, their semiconducting nature needs to be addressed for application in electrocatalysis. Transition metal nitrides and carbides, and 2D layered materials such as MXenes are also interesting as alternative supports in electrocatalysis.

Despite advancements made in the last couple of decades, there is still significant potential for breakthroughs in the field of electrocatalysis. For instance, the dependence of fuel cells and water electrolyzers on PGMs, particularly Pt and Ir, needs to be addressed to utilize these carbon-neutral technologies on a large scale. This can be achieved by optimizing PGM-based electrocatalysts (both active sites and integration of advanced supports) and developing methods for efficient recycling of these critical raw materials, or by tailoring non-PGM-based catalysts with comparable activity and durability.
